# *Trypanosoma cruzi* Necrotizing Meningoencephalitis in a Venezuelan HIV^+^-AIDS Patient: Pathological Diagnosis Confirmed by PCR Using Formalin-Fixed- and Paraffin-Embedded-Tissues

**DOI:** 10.1155/2014/124795

**Published:** 2014-12-29

**Authors:** Marcello Salvatore Rossi Spadafora, Ghislaine Céspedes, Sandra Romero, Isabel Fuentes, Alpidio A. Boada-Sucre, Carmen Cañavate, María Flores-Chávez

**Affiliations:** ^1^Unit of Toxoplasmosis and Intestinal Protozoosis, Department of Parasitology, National Centre for Microbiology, Carlos III Health Institute, Carretera de Majadahonda-Pozuelo Km 22, Majadahonda, 28220 Madrid, Spain; ^2^Section of Research on Ultrastructural Pathology and Molecular Biology, José A. O'Daly Institute for Pathology, Faculty of Medicine, Central University of Venezuela, Caracas 1080-A, Venezuela; ^3^Section of Neuropathology, José A. O'Daly Institute for Pathology, Faculty of Medicine, Central University of Venezuela, Caracas 1080-A, Venezuela; ^4^Section of Nephropathology, José A. O'Daly Institute for Pathology, Faculty of Medicine, Central University of Venezuela, Caracas 1080-A, Venezuela; ^5^Laboratory of Electron Microscopy, Institute of Scientific and Technological Studies, Simón Rodríguez University, Caracas 1041-A, Venezuela; ^6^Unit of Leishmaniasis and Chagas Disease Diagnosis, Department of Parasitology, National Centre for Microbiology, Carlos III Health Institute, Carretera de Majadahonda-Pozuelo Km 22, Majadahonda, 28220 Madrid, Spain

## Abstract

Coinfections with human immunodeficiency virus (HIV) and infectious agents have been recognized since the early 90s. In the central nervous system (CNS) of HIV^+^ patients, parasitic protozoans like *Toxoplasma gondii* have been described as responsible for the space occupying lesions (SOL) developed. However, the involvement of *Trypanosoma cruzi* is also described but appears to be less frequent in acquired immunodeficiency syndrome (AIDS) and transplant recipients, associated with necrotizing myocarditis and neurological symptoms related to the occurrence of necrotizing pseudotumoral encephalitis (NPE) and meningoencephalitis (NME). The present work aims to present a Venezuelan case of NME associated with the coinfection of HIV and a *T. cruzi*-like trypanosomatid as well as its evolution and diagnosis by histopathological techniques, electron microscopy, and PCR methods using formalin-fixed- (FF-) and paraffin-embedded- (PE-) tissues. Postmortem cytological studies of leptomeninges imprints reveal the presence of trypomastigotes of *Trypanosoma* sp. Histopathological and electron microscopy studies allowed us to identify an amastigote stage and to reject the involvement of other opportunistic microorganisms as the etiological agent of the SOL. The definitive confirmation of *T. cruzi* as the etiological agent was achieved by PCR suggesting that the NME by *T. cruzi* was due to a reactivation of Chagas' disease.

## 1. Introduction

In tropical and subtropical populations worldwide, coinfections with human immunodeficiency virus (HIV) and infectious agents have been recognized since the early 90s. The gastrointestinal tract (GIT) and the central nervous system (CNS) are the main targets of opportunistic infections in patients with acquired immunodeficiency syndrome (AIDS) [1–4]. In this sense, GIT of HIV^+^ patients is a target for parasitic infections by* Cryptosporidium parvum*,* Isospora belli*,* Blastocystis* spp. [5, 6], microsporidians like* Septata intestinalis* [7] and* Enterocytozoon bieneusi* [8],* Entamoeba histolytica/dispar* complex,* Entamoeba coli*,* Giardia lamblia*,* Endolimax nana*,* Cyclospora cayetanensis*,* Chilomastix mesnili*, and* Leishmania* sp. [9, 10], and helminths like* Ascaris lumbricoides*,* Trichuris trichiura*,* Strongyloides stercoralis*, and* Hymenolepis nana* [6], with common symptoms such as weight loss, diarrhoea, and abdominal pain.

In the CNS, mycotic agents like* Histoplasma capsulatum* [11] and* Cryptococcus neoformans* [12] as well as mycobacteria belonging to* Mycobacterium tuberculosis* [13],* M. avium* complex,* M. abscessus* complex, and* M. kansasii and M. massiliense* species [14] and parasitic protozoans like* Toxoplasma gondii* [2] have been described as responsible for the space occupying lesions (SOL) in HIV^+^-AIDS patients. However, involvement of microsporidian (*Enterocytozoon bieneusi* and* Septata intestinalis*) and trypanosomatids like Chagas' disease agent (*Trypanosoma cruzi*) is also described but appears to be less frequent among transplant recipients and AIDS patients [15].

Because of pandemic nature of HIV infection, AIDS is spreading to regions where Chagas disease is endemic [15], taking place in either primo-infections between HIV^+^ patients or reactivation of subclinical infections with (i) sporadic gastrointestinal manifestations characterized by atrophic intestinal villi and inflammatory lesions of* lamina propria*, (ii) heart manifestations associated with necrotizing myocarditis, and (iii) neurological symptoms related to the occurrence of necrotizing pseudotumoral encephalitis (NPE) and meningoencephalitis (NME) [16].

Statistics performed for Latin America have been reported that there are currently between 15 and 16 million Chagasic patients in any of its phases (acute, indeterminate, or chronic) and 75–90 million at risk [17]. Furthermore, there are 1.4 million HIV-positive patients with or without clinical manifestations of AIDS [18]. Taking in account this as well as the geographical overlap of the two diseases and the migratory movements of populations, the association between Chagas encephalitis and HIV-AIDS should be taken into account not only in the early diagnosis and proper treatment, given their poor prognosis, but also in routine pathological diagnosis. In this regard, the development of NPE and NME in AIDS constitutes a serious problem of pathological diagnosis, because this kind of lesions may be produced by opportunistic protozoan parasites such as* T. gondii* and* T. cruzi* but also by mycobacteria, fungi, and neoplastic diseases like non-Hodgkin's lymphoma [19, 20], progressive multifocal leukoencephalopathy (PML) [19, 21], astroblastomas, astrocytomas, and glioblastoma [22]. Furthermore, they are very difficult to differentiate clinically and at level of neuroimaging. In the present work, we describe a fatal case of an opportunistic infection of the CNS of a Venezuelan HIV^+^ patient, firstly treated as a case of cerebral toxoplasmosis and finally postmortem confirmed by histopathology and transmission electron microscopy and for the very first time in Venezuela by PCR from FF- and PE-CNS-tissues.

## 2. Material and Methods

### 2.1. Ethics Statement

The research that is reported in this paper has been performed according to the statements of the Instituto Anatomopatológico José A. O'Daly and the Instituto de Salud Carlos III and in agreement with the Helsinki Declaration. In this regard, all of the research meets the ethical guidelines, including adherence to the legal requirements of the study country.

### 2.2. Case Presentation

A male patient of 38 years old, born in a rural area of the Trujillo state (Venezuela), where the seroprevalence to* T. cruzi* was 19.2–23.8% and the main triatomine vector was* Rhodnius prolixus* [23] came to the emergency unit of the Caracas University Hospital with fever, generalized seizures, and progressive neurological impairment (day 0), whose symptoms appeared a week before admission (day 7). The patient was living in La Guaira city (Vargas state, Venezuela) where the mortality rate due to HIV-AIDS was the second highest of Venezuela in 2009 (10.5 deaths × 100,000 inhabitants) [24] and an outbreak of oral Chagas disease occurred in Chichiriviche de la Costa in the same year, affecting 54 children and causing 3 deaths by acute heart failure [25].

Patient also reported to be bisexual with a promiscuous lifestyle and to have an HIV^+^ diagnosis. The physical examination revealed a body temperature of 38-39°C and a CD4^+^ T-lymphocytes count of <200 cells/mm^3^ (<14%) (AIDS C_3_ stage). According to the National Program of AIDS and Sexually Transmitted Infections of the Ministry of Health, the patient should have been treated with antiretrovirals, but, due to difficulties in obtaining these drugs, the patient was not following a retroviral therapy. The patient was hospitalized and a computed tomography (CT) of head was done revealing two rounded SOL ring-shaped in the frontal region and basal nuclei (CT images not shown). As a consequence of neuroimaging results, a presumptive diagnosis of toxoplasmosis was given to the patient, for which he received an empirical treatment with 200 mg of pyrimethamine in the first day and 75 mg/day since the second day, in combination with 100 mg/kg of body weight/day (4–6 g/day) of sulphadiazine (day 1). Since patient did not show improvement of his health condition, a lumbar puncture was done to obtain samples of cerebrospinal fluid (CSF) (day 4) which were cytologically studied. Because results from CSF cytology showed few parasitic flagellate protozoans morphologically compatible with trypomastigotes of* Trypanosoma* sp., the treating physicians consulted the departments of immunology and tropical medicine in order to confirm the diagnosis and to modify the treatment.

The characteristic morphology of flagellates and the epidemiological nexus of patient in Trujillo state (a Chagas' disease endemic area), without a specific diagnosis, allowed physicians not only to reformulate the case as probable infection of the CNS by* Trypanosoma cruzi* but also to start a specific treatment with Nifurtimox (6 mg/kg of body weight/day for 60 days in two daily fractions) due the absence of Benznidazole (day 7). The patient had a torpid evolution, without a favourable response to treatment, and died 3 days after the treatment beginning (day 10). Autopsy was done 1 hour after his death, according to the standards protocols at José A. O'Daly Institute for Pathology (Faculty of Medicine, Central University of Venezuela), in order to perform the corresponding pathological studies and confirmation of the pathological diagnosis at electron microscopical and molecular level by PCR.

### 2.3. Pathologic Studies

After the autopsy, CNS samples from five nodular lesions with a necrotized centre surrounded by a white halo were prepared for pathological analysis by conventional histological techniques. In this regard, a total of about 40 sections of 5–7 *μ*m thick from areas of injury as well as leptomeninges imprints were stained with Hematoxylin/Eosin, Giemsa, Ziehl-Neelsen, and Grocott. A total of 10 sections 3 *μ*m thick were used for the detection of HIV, cytomegalovirus (CMV), and the protein of 30 kDa (p30) of* T. gondii* by immunohistochemistry with specific monoclonal antibodies. Additionally, PE-tissues of the CNS from areas of injury were deparaffinised and prepared for transmission electron microscopy (TEM) according to Nasr et al. [26] technique. A total of 10 ultrathin sections were cut with a diamond knife in a Porter-Blum MT2-B ultramicrotome and stained with uranyl acetate and lead citrate. Sections were examined with a Hitachi H-500 transmission electron microscope operated at 100 kV.

### 2.4. Molecular Studies

#### 2.4.1. DNA Extraction

Seven to twelve sections (5 *μ*m thick) from samples of CNS formalin-fixed and paraffin-embedded were deparaffinised according to Coombs et al. [27] technique. Briefly, sections were recovered in vials containing 100 *μ*L of 0.5% Tween 20, vortexed, and subjected to 3-4 cycles of heating (56°C for 10 minutes) and cooling in an ice bath for 2 minutes and then centrifuged at 14860 ×g for 15 minutes. The paraffin layer (disc-shaped) formed at the surface was removed using a needle. Tissues recovered in the pellet were digested for 3 hours at 55°C with 100 *μ*L of NET-10 digestion buffer (10 mM Tris-HCl pH 8.0, 100 mM NaCl, 1 mM EDTA) containing 8 *μ*L of Proteinase K (10 mg/mL) and 50 *μ*L of 10% SDS. DNA was purified from extract using Chelex 100 technique [28] and precipitated with isopropyl alcohol containing 2 *μ*L of glycogen (2 mg/mL). The resulting pellet was washed at 4°C by centrifugation (14860 ×g for 5 minutes) with cold (−20°C) 70% ethanol. Finally, DNA was dried out by low heating in a speed vacuum device, heated at 95°C for 15 minutes, and suspended with 50 *μ*L of sterile deionized water.

#### 2.4.2. Polymerase Chain Reaction (PCR)

Three PCR techniques were applied in order to detect the presence of* T. gondii* and* T. cruzi* in samples. For* T. gondii*, a* nested*-PCR for the amplification of sequences from B1 gene was applied [29], while, for* T. cruzi*, two PCR techniques were used. The first one used the oligonucleotides 121-122 for the amplification of gene sequences of the minicircle variable regions from kinetoplast DNA [30], while the second one (with Tcz_1_-Tcz_2_ oligonucleotides) was designed for the amplification of repeated sequences of* T. cruzi* satellite DNA [31]. Strict methods were used to avoid contamination [32] and all of the assays included negative and positive controls. Amplicons generated were detected by electrophoresis in 2% agarose gels stained with ethidium bromide and its size is estimated using DNA markers.

## 3. Results

### 3.1. Autopsy Findings

The most important findings of the autopsy at macroscopic and general level were as follows.

 Respiratory tract: bilateral congestion of lungs and oedema, bronchopneumonia, bilateral fibrinous pleuritis, and bilateral hydrothorax.
 Cardiovascular system: Chagas cardiomyopathy (heart weight: 300 g).
 GIT: nonspecific chronic glossitis and Kaposi's sarcoma in stomach which was observed, as well as congestion and pancreatic haemorrhage.
 Liver: hepatomegaly (liver weight: 2250 g) with mild periportal, subcapsular steatosis, and acute congestion.
 Spleen: Kaposi's sarcoma, splenomegaly (spleen weight: 350 g) with fibrosis, splenic congestion, and depletion of the white pulp. Kaposi's sarcoma was also revealed in skin.
 Adrenal glands: necrotizing adrenalitis by cytomegalovirus (CMV).
 CNS: at macroscopic level, the vertical-transverse sections of brain showing the presence of five nodular lesions with a necrotized centre surrounded by a white halo. Three out of five nodules were located in the frontal lobes (one at the right and two at the left) and two in the occipital lobes. The size of the biggest one was 5.0 × 4.0 × 2.0 cm. In addition, moderate cerebral oedema (brain weight 1600 g) with rows of orbital compression, bilateral uncus, hyperaemia, and congestion of leptomeninges accompanied by purulent exudate were the most remarkable findings.

### 3.2. Cyto- and Histopathological Findings in CNS

**Figure 1:**
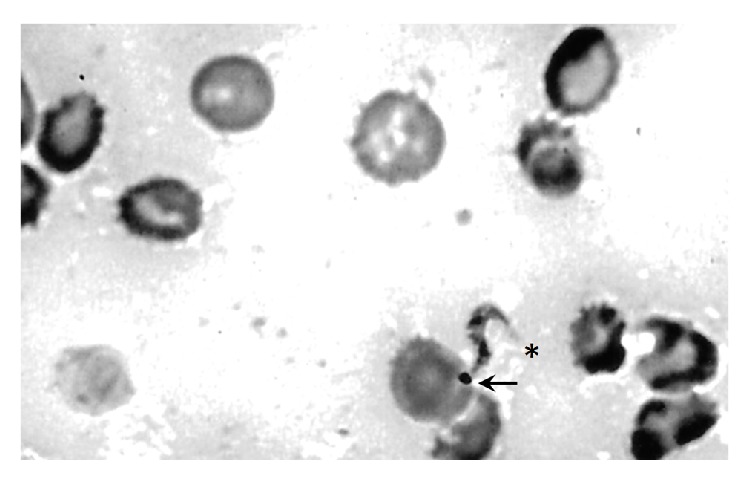
Cytology of leptomeninges imprints. Note the presence of an* S*-shaped trypomastigote compatible with the morphology of a* Trypanosoma* sp. (→) kinetoplast, (∗) free flagellum (Giemsa stain, ×400).

Figure 1 shows a microphotograph of the cytological findings from a Giemsa-stained leptomeninges imprints. Note the presence of an* S*-shaped trypomastigote, characterized by a prominent terminal kinetoplast at the pointed posterior end and a free flagellum at the anterior end.

**Figure 2:**
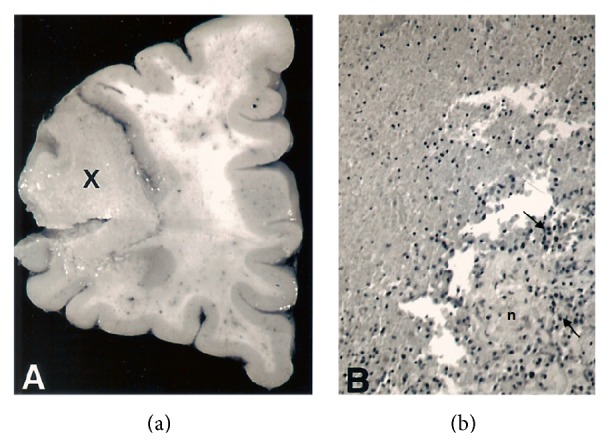
(a) Macroscopic view of a vertical-transverse section of the frontal lobe of brain. A space occupying lesion in the brain cortex and white matter is marked as X. Note the presence of punctate haemorrhages in grey and white matter. (b) Microphotograph of a vertical-transverse section of the lesion in (a) showing the histologic aspect of the expansive nodule (X). Note the presence of necrosis (n) of white matter with a perilesional inflammatory component (→) (H/E, ×100).

**Figure 3:**
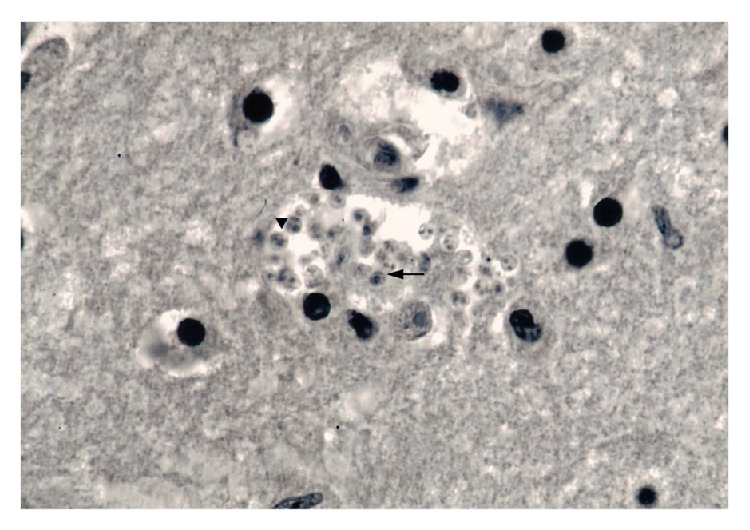
Gliomesenchymal cluster showing microorganisms of 1.5–3 μm wide with the morphology of amastigotes, (▼) free and (→) inside cytoplasm of a macrophage (H/E, ×400).

Figure 2(a) shows the expansive nature of a lesion in the biggest nodule of the frontal lobe of brain. In this sense, the lesion occupies the cortex and white matter. Histologically highlights the alteration of the architecture and the disorganization of the cerebral cortex and white matter with the corresponding glial scar. In addition, large areas of well-circumscribed necrosis with perilesional inflammatory component were observed (Figure 2(b)), as well as multiple areas of gliomesenchymal cells clusters containing intracellular microorganisms of 3-4 *μ*m in the cytoplasm of macrophages and glial cells (Figure 3).

Other histopathological findings in the cortex and white matter correspond to the presence of neurons with acute degeneration, Alzheimer type II glial cells, oedema and degeneration of the myelin sheath, reactive astrocytosis, lymphoplasmacytic inflammatory infiltrate in veins and arterioles wall and in perivascular location, foci of haemorrhage, and mixed glial proliferation and infiltration with macrophages in white matter. At level of the third ventricle were frequent small haemorrhages on the floor and walls as well as discontinuity of ependymal epithelium.

No parasites were observed in histological preparations of dura mater and leptomeninges, although we observed the presence of inflammatory infiltrate of lymphoplasmacytic nature and macrophages.

**Figure 4:**
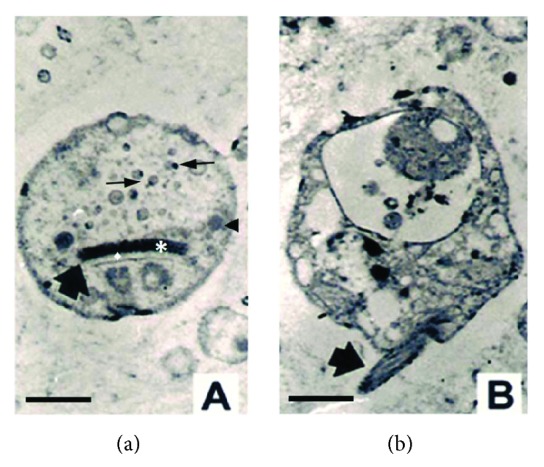
Transmission electron micrographs of intracellular microorganisms detected in the necrotizing space occupying lesion of the brain showed in Figure 4. Note the typical ultrastructure feature of a stercorarian amastigote. Figure 5(a) shows a transverse section of parasite with the discoidal kinetoplast (arrow) while Figure 5(b) shows the presence of a short flagellum or pseudoflagellum. In Figure 5(a), (∗) kinetoplast DNA, (♦) kinetoplast matrix, (→) acidocalcisomes, and (◄) glycosome. (Bar 0.4 μm.)

All of the histological sections were negative to mycobacteria or nocardias (Ziehl-Neelsen),* Histoplasma* sp. and* Cryptococcus neoformans* (Grocott) and* T. gondii*, but positive to CMV in adrenal cells and HIV in lymphocytes (results not shown). Transmission electron microscopy shows the presence of intracellular amastigotes (Figure 4), highlighting the presence of parasites with a pseudoflagellum and a well-developed and prominent kinetoplast (0.5 *μ*m) as a slightly concave disk with a very electron-dense nucleoid, separated from its envelope or lipid bilayer by a variable amount of a matrix with low to moderate electronic density.

### 3.3. Molecular Studies

**Figure 5:**
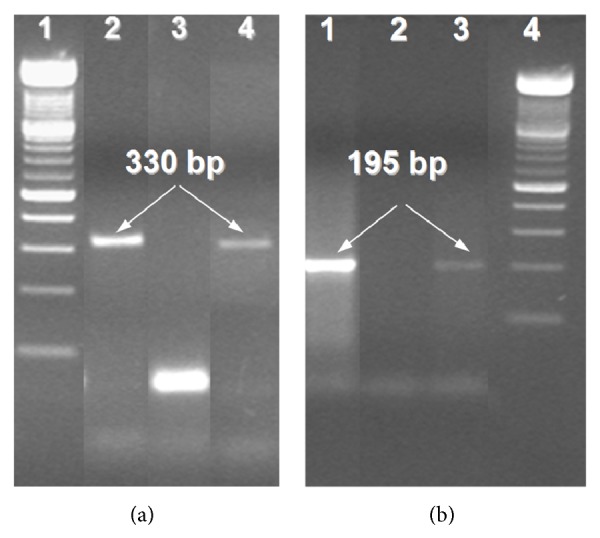
Molecular detection of* T. cruzi* by PCR. (a) Amplification of the variable region of kDNA minicircles from trypanosomes (amplicon: 330 bp); line 1: 100 bp DNA ladder as molecular marker; line 2: positive control of DNA from human blood infected with* T. cruzi*; line 3: distilled water as negative control; and line 4: DNA from PF- and PE-sections of the frontal lobe SOL. (b) Amplification of a repeated sequence from* T. cruzi* satellite DNA (amplicon: 195 bp); line 1: positive control of DNA from human blood infected with* T. cruzi*; line 2: distilled water; line 3: DNA from PF- and PE-sections of the frontal lobe SOL; and line 4: 100 bp DNA ladder as molecular marker.

**Figure 6:**
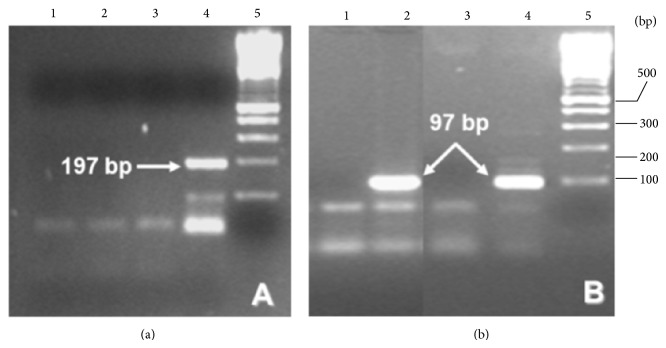
Molecular detection of* T. gondii* by* nested*-PCR of a B1 gene sequence. (a) First round PCR and (b)* nested*-PCR. Line 1: DNA from PF- and PE-sections of the frontal lobe SOL; line 2: DNA from PF- and PE-liver sections from a* T. gondii* experimentally infected mice; line 3: distilled water as a negative control; line 4: DNA from tachyzoites of* T. gondii* (RH strain); and line 5: 100 bp DNA ladder as molecular marker.

Results of specific amplification of trypanosome and* T. cruzi* DNA sequences (Figure 5(a)) clearly show the presence of an amplicon band of 330 bp corresponding to the amplification of minicircle kinetoplast DNA of trypanosomes. However, an accurate molecular identification of amastigotes from CNS lesions was achieved with a PCR technique designed for the amplification repeated sequences of satellite DNA. As it can be shown in Figure 5(b), an amplicon band of 195 bp specific for* T. cruzi* was obtained. The absence of* T. gondii* as microorganism responsible for SOL was also confirmed by the absence of the B1 gene amplicon of this apicomplexa protozoan in DNA extracted from PF- and PE-CNS-tissues (Figure 6).

## 4. Discussion

Since several decades,* T. cruzi* has been added to the extended list of infectious agents and parasitic protozoans responsible for opportunistic infections in AIDS patients. In that respect, classical syndromes associated with Chagas' disease and AIDS, namely, meningoencephalitis, cardiomyopathy, and megaesophagus, are well known [15, 33–35]. Those related to intestinal infections [16] that, until about 25 years ago, were unknown have been added to the extended list of pathogens [36, 37].

In the case presented in this work, as well as in others submitted by the Caracas University Hospital (Venezuela) to our laboratory, the neurologic involvement with tomographic lesions similar to those exhibited by cases of toxoplasmosis in HIV^+^ patients, together with the absence of specific parasitological, serological, and molecular tests in the laboratory facilities of the hospital, determined the establishment of an empirical treatment for* T. gondii*, a very common behaviour in the management of HIV^+^ patients in the intensive care units of our country.

The therapeutic failure of the empirical treatment for* T. gondii* established on day 1 correlates with the results obtained by immunohistochemistry and* nested*-PCR for the detection of* p*30 protein and B1 gene of* T. gondii* in CNS-tissues, respectively.

The presence of* T. cruzi* in SOL of the CNS such as necrotizing pseudotumoral encephalitis and meningoencephalitis is a rare complication of Chagas' disease observed only in immunosuppressed patients and it has been associated with reactivation of the disease [15]. The development of necrotizing SOL during HIV infection is a problem of pathological diagnosis, due to the difficulties arising in the differential diagnosis with neoplastic diseases and infections by other opportunistic CNS agents like* T. gondii*.

Despite its low sensibility, the first approach in the laboratory diagnosis of Chagas disease in HIV^+^ patients depends on the microscopic demonstrations of* T. cruzi* trypomastigotes in samples of blood and CSF. Alternatively, when the infectology department of the hospital has access to technological resources, serological and molecular techniques for the detection of* T. cruzi* antigens or genes, respectively, are very useful.

The second approach could not be implemented due to the absence of ELISA or PCR based techniques in the facilities of the hospital and unfortunately the first one was not applied since day 7, moment in which trypomastigotes forms of a* Trypanosoma* sp. were detected on CSF samples taken at day 4, as a consequence of the failure in the treatment against* T. gondii*. As a consequence of these facts, specific treatment with Nifurtimox was applied, and 3 days after that, CSF samples were taken and probably the delay in the reorientation of the case was determining in the patient death.

Although the diagnosis of cerebral Chagas as a probably cause of death was suggested by the University Hospital of Caracas, the aim of the pathologic studies presented here was not only to establish the alterations responsible for the death, but also to confirm the involvement of* T. cruzi* by amplification of specific gene sequences by PCR and to exclude the participation of other opportunistic pathogens.

In this regard, histological staining of Grocott and Ziehl-Neelsen helped us to discard the mycotic and mycobacterial involvement, respectively. In addition, pathologic studies allowed us to detect trypomastigotes and amastigotes of* Trypanosoma* sp. in the SOL of the CNS according to cytological (Giemsa-stained imprints of leptomeninges), histopathological (Hematoxylin-Eosin stained sections of SOL), and ultrastructural criteria. The cytology results of leptomeninges imprints show the presence of few trypomastigotes forms, while the histopathological studies revealed an abundance of intracellular and free amastigotes in the necrotizing SOL.

The involvement of* T. gondii* as responsible for the pathology was discarded because of the absence of cysts and free tachyzoites in the CNS, as it could be demonstrated in tissue sections stained with H/E and in immunoreactions using specific monoclonal antibodies to* T. gondii p*30 protein (results not shown). Furthermore, these results were confirmed by the absence of B1 gene amplicons by the* nested*-PCR applied. Immunohistochemistry also allowed us to confirm the positivity of white blood cells to HIV in the CNS of the deceased, as well as the detection of CMV (result not shown) as the etiological agent causing the adrenalitis described.

The contribution of TEM in our work was to confirm the parasitological findings done with the light microscope, based on the ultrastructural features of the trypanosome intracellular stage (amastigote). In this regard, the amastigote multiplying stage developed inside glial cells and macrophages of the necrotizing SOL, showing a kinetoplast type A [38] as well as other characteristic features of amastigotes of* T. cruzi* like the pseudoflagellum. However, the species-specific confirmation of* T. cruzi* as the protozoan responsible for the necrotizing SOL was achieved by PCR using specific primers directed to the amplification of the variable regions of trypanosomes kDNA minicircles (amplicon: 330 bp) [30] and repeated sequences of satellite DNA of* T. cruzi* (amplicon: 195 bp) [31].

According to epidemiological data and clinical information, it is very likely that* T. cruzi* infection would be acquired in the Trujillo state (an endemic area) and evolved as an asymptomatic or subclinical infection. After this primo-infection, the disease progressed to the undetermined and chronic phase. In this regard, between 10 and 30% of seropositive people to* T. cruzi* evolve to a chronic asymptomatic stage of 10–20 years length [15, 39]. The features of this stage include myocarditis and destruction of the cardiac conduction system, facts sustained by the pathologic diagnosis of Chagasic cardiomyopathy done in this case. In addition, it is a very well described fact that Chagasic myocarditis (considered the second most common presentation in HIV^+^ patients) can coexist in some patients with meningoencephalitis [40].

The migration of the patient from the rural area of origin in the Trujillo state to the Vargas state (where the incidence of HIV-AIDS is higher) together with the adoption of a sexual risk behavior determined the HIV coinfection, the subsequent immunosuppression, and the infection with CMV. This idea constitutes a very well documented fact; in this sense, Corti [41] and Cordova et al. [40] refer that, in* T. cruzi*-HIV coinfection, people generally acquire* T. cruzi* infection while they are living in rural endemic areas, years before HIV infection. In addition, the international travels and migrations between rural endemic areas and urban nonendemic areas contribute to HIV infection [42, 43]. Finally, the immunosuppression accumulated for years and the difficult access to antiretroviral therapy favours the transient rise in* T. cruzi* parasitaemia from cryptic to higher levels, causing injuries in different organ systems and neurologic symptoms in 70–85% of cases [40], cardiovascular symptoms in 10–55% [39], and gastrointestinal symptoms in lesser extent [16, 44].

## 5. Conclusions

The original contribution of our work is the species-specific confirmation of the coinfection of HIV and* T. cruzi* in the CNS of an HIV^+^ patient using, for the very first time in Venezuela, DNA extracted from PF- and PE-tissues and specific PCR assays to amplify* T. cruzi* and* T. gondii* gene sequences. In addition, the work represents the first evidence in our country for the adoption of molecular techniques to confirm the pathologic diagnosis of deaths caused by these protozoan parasites. From the pathologist points of view and in spite of the disadvantages of using pathological samples for DNA extraction and gene amplification by PCR because of (i) DNA fragmentation, (ii) crosslinking of DNA with cell proteins through hydroxymethylene bridges, (iii) chemical modification of DNA nucleotides, or (iv) inhibition of polymerases by heavy metal traces present in organic (ethanol and xylene) solvents (technical grade) used in pathological routines [45, 46], the adoption of PCR techniques specially in the real time format could be turned in very useful tools when conventional stainings, immunohistochemical reactions, and TEM fail in the confirmation of the etiology of the death.

Furthermore, the adoption of these molecular approaches could result in very significant benefits in epidemiological retrospective studies and in the knowledge of the genotypes responsible for the recent Chagas disease outbreaks described in Venezuela. Actually our group is working on the optimization of techniques for DNA and RNA extraction as well as in the implementation of real time PCR techniques for the confirmation of deaths due to not only protozoan parasites (*T. cruzi*,* T. gondii*, and* Plasmodium* sp.) but also mycobacteria, spirochetes (*Leptospira* sp.), and arboviruses (yellow fever and dengue fever virus).
